# Potential implications of SARS-CoV-2 oral infection in the host microbiota

**DOI:** 10.1080/20002297.2020.1853451

**Published:** 2020-11-29

**Authors:** Zhenting Xiang, Hyun Koo, Qianming Chen, Xuedong Zhou, Yuan Liu, Aurea Simon-Soro

**Affiliations:** a Biofilm Research Labs, Levy Center for Oral Health, Department of Orthodontics, Divisions of Pediatric Dentistry and Community Oral Health, School of Dental Medicine, University of Pennsylvania, Philadelphia, Pennsylvania, USA; b State Key Laboratory of Oral Disease & Human Saliva Laboratory & National Clinical Research Center for Oral Diseases, West China Hospital of Stomatology, Sichuan University, Chengdu, Sichuan, China; c Center for Innovation & Precision Dentistry, School of Dental Medicine, School of Engineering and Applied Sciences, University of Pennsylvania, Philadelphia, Pennsylvania, USA; d Stomatology Hospital, Zhejiang University School of Medicine, Zhejiang University, Hangzhou, Zhejiang China; e Department of Stomatology, School of Dentistry, University of Seville, Seville, Spain

**Keywords:** Severe acute respiratory syndrome coronavirus 2, angiotensin-converting enzyme 2 (ACE2), oral-gut axis, oral-lung axis, microbiota

## Abstract

The oral cavity, as the entry point to the body, may play a critical role in the pathogenesis of SARS-CoV-2 infection that has caused a global outbreak of the coronavirus disease 2019 (COVID-19). Available data indicate that the oral cavity may be an active site of infection and an important reservoir of SARS-CoV-2. Considering that the oral surfaces are colonized by a diverse microbial community, it is likely that viruses have interactions with the host microbiota. Patients infected by SARS-CoV-2 may have alterations in the oral and gut microbiota, while oral species have been found in the lung of COVID-19 patients. Furthermore, interactions between the oral, lung, and gut microbiomes appear to occur dynamically whereby a dysbiotic oral microbial community could influence respiratory and gastrointestinal diseases. However, it is unclear whether SARS-CoV-2 infection can alter the local homeostasis of the resident microbiota, actively cause dysbiosis, or influence cross-body sites interactions. Here, we provide a conceptual framework on the potential impact of SARS-CoV-2 oral infection on the local and distant microbiomes across the respiratory and gastrointestinal tracts (‘*oral-tract axes*’), which remains largely unexplored. Studies in this area could further elucidate the pathogenic mechanism of SARS-CoV-2 and the course of infection as well as the clinical symptoms of COVID-19 across different sites in the human host.

## Introdution

The global threat of COVID-19 has spurred research efforts on an unprecedented scale and pace to advance the knowledge about infection, transmission, and early detection of SARS-CoV-2 [1]. Yet, the oral cavity, as a port-of-entry and exit, remains an overlooked interface to further understand the infection mechanisms of SARS-CoV-2 and its impact in the oral and systemic health. Detection of the virus in saliva and the availability of oral tissues enriched with ACE2 receptors [2] indicate that the oral cavity can be an important reservoir of SARS-CoV-2, which may serve as an entry point to the respiratory and gastrointestinal tracts, influencing both infection and clinical symptoms. Patients infected with SARS-CoV-2 appear to have high accumulation of pathogenic oral bacteria, where dysbiosis in the oral microbiome could influence the severity of respiratory symptoms and gastrointestinal manifestations [3,4]. Hence, the oral cavity could play an important role for the establishment of infection by SARS-CoV-2 and the severity of COVID-19 complications. Here, we focus on the potential impact of oral infection of SARS-CoV-2 to the oral, lung, and gut microbiomes. The study of ‘*oral-tract axes*’ may lead to a more integrated conceptual framework to further understand the etiopathogenesis of COVID-19 and help develop accurate diagnostics and precise therapeutics to curb this global crisis.

## Potential oral reservoirs of SARS-CoV-2

In the ongoing COVID-19 pandemic, recurrence of clinical signs and symptoms after recovery are emerging in infected patients. Retrospective data have also shown the possibility of SARS-CoV-2 reactivation [5]. Moreover, viral shedding in asymptomatic individuals and recovered patients after the cessation of respiratory symptoms has been documented [6,7]. These observations have risen questions on whether SARS-CoV-2 could persist in certain anatomical reservoirs that act as a source of subsequent disease in an active or latent state. In addition, it is possible that the presence of tissue reservoirs in the oral cavity can cause biological alterations locally and distally leading to exacerbated disease complications and delayed recovery time.

The infectivity of SARS-CoV-2 depends on the ability of this virus to enter the cells, and there is a clear evidence that ACE2 is the primary receptor interacting with virus spike protein when entering into the cell [8]. Since the oral cavity is one of the first entry points to the body, there is a high potential that this pathway of viral infection and colonization is critical for the onset of COVID-19 [2,9]. Transcriptome data analysis by Song et al. showed that ACE2 and TMPRSS2 were expressed in salivary glands [10]. A previous study has indicated that salivary gland epithelial cells show elevated expression of ACE2 [11]. For example, the ACE2 expression in minor salivary glands was greater than that in the lungs indicating that salivary glands could be an important site for SARS-CoV-2 infection. Supported evidence showed higher expression of SARS-CoV-2 in critically ill patients which suggested high viral loads or dysfunctional salivary glands at the late stage of the infection [12]. Furthermore, SARS-CoV-2 might induce acute sialadenitis and associated symptoms such as pain, inflammation, and secretory dysfunction in salivary glands [13,14]. In addition to a high rate of SARS-CoV-2 detection in saliva [15], a recent publication has reported that salivary SARS-CoV-2 positivity in a patient who was in convalescence [16]. Xu et al. stated that the spread of COVID-19 through asymptomatic infection may come from the contaminated saliva secreted by infected salivary glands which possibly serve as a potential reservoir for SARS-CoV-2 [9], however, more evidence is required [17].

ACE2 also appears to be expressed on the oral mucosa as determined through public bulk-seq RNA datasets [2]. Among the various clinical symptoms, SARS-CoV-2 positive patients often complain of pain in tongue and gustatory dysfunction including loss of smell and taste [18], which may be associated with the higher expression of ACE2 in the epithelial cells of the tongue or the possibility of direct neuron or glia infection by SARS-CoV-2 [19]. However, the mechanism of how primary infection of non-neural cells alters chemical perception needs further investigation. In addition, oral mucosal lesions were described in SARS-CoV-2 positive patients [20]. Moreover, enriched expression of ACE2 sheddases (ADAM17 and ADAM10) and endopeptidases (CAPN1 and CAPNS1) indicated the higher membrane fusion activity of the virus in the different sites of oral mucosa [21], suggesting it could be a reservoir for the virus.

**Figure 1.**
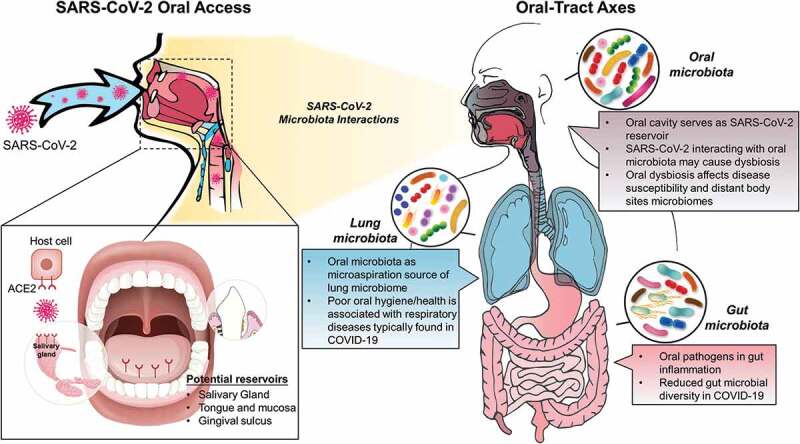
A conceptual framework of the impact of SARS-CoV-2 oral infection on the local and distant microbiomes across the ‘oral-tract axes’. The left panel illustrates the potential reservoirs of SARS-CoV-2 during the infection of the oral cavity. The right panel highlights current knowledge and potential interactions of the virus across the oral/gastrointestinal and respiratory tracts, which remain largely unexplored

Another potential niche for SARS-CoV-2 is the gingival sulcus, a well-established microbial niche where enzymes and inflammatory molecules are released promoting the colonization of microorganisms [22]. Herpes simplex virus, Epstein-Barr virus, human papilloma virus, and human cytomegalovirus have all been detected in both clinically healthy gingival sulci and periodontal pockets [23]. These observations of high viral proliferation in the gingival sulci may be due to a symbiotic relationship between microorganisms manifesting in these crevices and the virus [24]. Moreover, gingival crevicular fluid (GCF) has been speculated to harbor SARS-CoV-2 released from infected periodontal cells or by terminal capillary complexes in periodontal tissues, which can subsequently reach the oral cavity by mixing with saliva [25,26]. However, technical limitations of isolating GCF for accurate measurements make validation of this possibility challenging [27]. Taken together, the oral cavity can be a significant reservoir of SARS-CoV-2 (Figure 1) that may impact the host both locally and across the interconnected body sites, particularly the upper respiratory and the lower gastrointestinal tract.

## Can SARS-CoV-2 oral infection impact local and distant microbiomes?

Since every surface of the human body exposed to the environment is colonized by a diverse microbial community, which is integrally tied to the human health, it is unsurprising that viruses have interactions with the host microbiota. Early evidence has suggested the relationship between microbial diversity of the lung microbiota and viral infection [28]. Likewise, the local microbiome could be disturbed by viral infection, such as SARS-CoV-2, resulting in dysbiotic communities [29]. Previous work using an unbiased high-throughput sequencing method to analyze the oropharyngeal microbiota of pneumonia patients with or without influenza virus infection has found a significant increase in the number of *Pseudomonas* and *Bacillus* species after influenza virus infection, whereas the number of *Prevotella, Veillonella*, and *Neisseria* decreased significantly [30]. Recently, the microbiota in the nasopharynx was also found to be altered in COVID-19 patients, where *Fusobacterium periodonticum* significantly decreased [31]. However, further investigations are needed to determine whether SARS-CoV-2 oral infection can cause local microecological imbalance and dysbiosis. Moreover, SARS-CoV-2 infection could predispose patients to coinfections and superinfections, leading to increased disease severity and mortality [32]. Opportunistic infections and complications caused by bacterial and fungal coinfection may have implications in COVID-19 diagnosis and treatment [33,34]. Studies have further revealed that not only respiratory viruses, but systemic infectious viruses can coinfect with SARS-CoV-2 [35,36].

Conversely, the microbiota can indirectly mediate colonization of non-indigenous microorganisms and subsequent infection by stimulating host responses such as mucosal immune defenses [37]. Therefore, resident microbiota dysbiosis caused by aging or systematic diseases may change the susceptibility and/or outcomes of COVID-19 [29]. Furthermore, disruptions in the structure and function of the microbiota can change the homeostasis between resident microbes and the host as well as the microenvironment and availability of nutrients, greatly altering infection susceptibility to the viruses [38]. However, the relationship between oral microbiota and susceptibility to SARS-CoV-2 infection remains poorly understood and requires further investigation both clinically and using available animal models.

In addition to localized microbiota-virus interaction in the oral cavity, the dysbiotic microbiota or the virus itself may also affect microbial activity in distal organs. Anatomically, the oral cavity is the shared connection between the gastrointestinal and the respiratory tracts (Figure 1). Microbiologically, the mouth is a highly diverse niche containing several bacterial species that can be also be found in the gut and lung suggesting connected body sites microbiomes [39]. For example, periodontal pathogens and immune response in periodontitis were recently implicated in the development of gut inflammation [4]. In addition, poor oral health and accumulation of pathogenic bacteria have been associated with respiratory diseases such as pneumonia [22], where oral species can be found in the lung microbiota of COVID-19 patients [3,40]. Therefore, it is plausible to consider whether the oral infection by SARS-CoV-2 and its impact in the local and distal microbiota can play significant roles in the severity of the infection and its clinical outcomes.

## Oral-gut axis: oral cavity and the gastrointestinal tract

The oral-gut axis is a metabolic and anatomical connection where the oral cavity is the first organ of the gastrointestinal tract. The environment of each organ integrating the digestive system harbors a related but distinctive microbiota across the tract [41]. In healthy individuals, oral bacteria can be found in the microbiota of the large intestine, suggesting the shedding of bacteria from the oral microbiome as a source for the lower gastrointestinal tract [42]. In gnotobiotic mice, human oral microbiota-associated species could successfully colonize the gut in the mice, suggesting a potential connection from oral to gut microbiota [43]. In lower gastrointestinal diseases, the gut microbiota previously linked to specific pathological conditions (e.g. colorectal cancer, rheumatoid arthritis, and inflammatory bowel disease) was correlated with oral microbiome dysbiosis or presence of oral bacteria in the gut [44–46].

Recent findings indicated the impact of the oral microbiota in intestinal inflammatory diseases, suggesting oral bacteria might translocate and migrate to the lower digestive tract exacerbating gut inflammation. Oral bacteria such as *Fusobacteriaceae, Pasteurellaceae*, and *Veillonellaceae* could be found in the intestine in inflammatory bowel disease [47]. Furthermore, commensal oral pathobionts (*Klebsiella*/*Enterobacter* species) can colonize ectopically the intestine activating the inflammasome, promoting colitis in an *in vivo* disease model [4]. We have recently found that specific oral bacteria can translocate to lower gastrointestinal organs in an animal model following disruption of the oral microbiome caused by an antimicrobial treatment, showing that local disturbances can cause microbiota changes at distant sites (Simon-Soro et al., unpublished).

In COVID-19 patients, bacterial and fungal opportunistic pathogens have shown increased levels in the gut microbiome [48], suggesting that the integrity of the gut microbiota could be disturbed by SARS-CoV-2, resulting in a dysbiotic community [29]. Consistent with this concept, a cross-sectional study revealed that the intestinal bacterial diversity is significantly reduced in COVID-19 patients, with higher relative abundance of opportunistic pathogens and lower relative abundance of beneficial symbionts [49]. Understanding how SARS-CoV-2 infection might impact the oral and gut microbiota not only locally but in distant connected body sites as well as their microbiome interactions could lead to new knowledge about COVID-19 pathogenic mechanisms.

## Oral-lung axis: oral cavity and the respiratory tract

The oral-lung axis through the upper respiratory tract connects oral and lung body sites [50]. Oral and nasal microorganisms are found as the main source of the lung microbiome. In healthy subjects, microaspirated oral bacteria composition have been found to be similar to a lung microbial community enriched in *Prevotella* or *Veillonella* [51]. In disease, we found lung abnormalities related to oral microbiome dysbiosis, suggesting the oral cavity as the microbial gateway for the lower respiratory tract [52]. Moreover, respiratory pathogens found in the oral cavity could reach the lung by microaspiration contributing to respiratory diseases as reported in acutely ill hospitalized patients [50]. Poor oral hygiene has been linked to the presence of opportunistic pathogens in other body sites, while oral diseases such as periodontitis has been strongly associated with asthma and pneumonia [22]. Previous studies also indicated that poor oral hygiene leading to oral microbial dysbiosis can accelerate lung function decline [53] and increase the incidence of pneumonia [54]. Data analysis overlapping bacterial pathogens in the oral cavity and lung in ventilator-associated pneumonia suggested the tongue microbiota as a putative reservoir [55]. In cystic fibrosis (CF), a correlation between *Pseudomonas aeruginosa* in subgingival plaque and its detection in the lung has been previously reported, suggesting that the mouth could be a gateway for the colonization of the pathogen in CF patients [56]. Conversely, the oropharyngeal microbiota in patients after lung transplantation showed severe dysbiosis community-wise with reduction of diversity and richness increasing anaerobic facultative bacteria. This suggested that lung intervention causes disturbances in the oral microbiota [52]. This bidirectional connection may be relevant in COVID-19 as opportunistic pathogens found in the oral cavity such as *Prevotella, Veillonella,* and *Capnocytophaga* have been identified in the lungs of patients infected by SARS-CoV-2 [40,57]. Periodontal-associated cytokines might drive the alteration of the respiratory epithelium via the aspiration of oral pathogens into respiratory organs to promote the adhesion of the virus. Therefore, the oral microbiome might impact lung infection and microbial colonization by SARS-CoV-2 [58].

In summary, we highlighted available data and provided a conceptual framework about potential implications of SARS-CoV-2 infection on the oral microbiota and its connection with the lung and gut microbiomes (Figure 1). The oral cavity appears to be a rich reservoir of SARS-CoV-2 and could play an important role in altering the interaction of local and distant microbiomes in the ‘*oral-tract axes*’ influencing the severity, clinical symptoms and transmission of COVID-19. Likewise, hundreds of microorganisms, including bacteria, viruses, and fungi, can be present in the mouth [59], which can also influence SARS-CoV-2 infection. However, it is unclear whether SARS-CoV-2 infection can: (i) alter the local homeostasis of the resident microbiota, (ii) actively promote dysbiosis, (iii) further disrupt an already unbalanced microbiota (e.g. caused by poor plaque control or inflammation), and/or (iv) influence cross-body sites interactions, requiring further clinical and mechanistic *in vivo* studies. Many outstanding questions remain unanswered: How does a dysbiotic oral microbiome impact SARS-CoV-2 entry? Is the oral cavity a reservoir or a latent niche for SARS-CoV-2? Is there a correlation between an oral dysbiotic microbiota and COVID-19 severity in infected patients? Could deleterious effects in saliva flow and composition due to disease or medication (e.g. Sjögren’s syndrome or antidepressants) impact SARS-CoV-2 oral infection and across the respiratory and gastrointestinal tracts? Further integration of metagenomics and clinical data might help characterize co-infections in COVID-19 and the connection between the oral microbiome and complications arising from the virus [3]. Given the importance of ‘*oral-tract axes*’ in COVID-19, multi-omics studies of clinical samples across the oral, lung, and gut sites could further elucidate the role of the host microbiota and its impact on SARS-CoV-2 infection (and vice-versa) and pathogenic mechanisms. Such integrative assessment may enhance the understanding of the course of infection and the clinical symptoms as well as reveal new ways to improve COVID-19 diagnosis, treatment, and prognosis to boost existing armamentarium to help control this global health crisis and societies to recover.
